# Specific immunotherapy in combination with *Clostridium butyricum* inhibits allergic inflammation in the mouse intestine

**DOI:** 10.1038/srep17651

**Published:** 2015-12-02

**Authors:** Yanhong Shi, Ling-Zhi Xu, Kangsheng Peng, Wei Wu, Ruijin Wu, Zhi-Qiang Liu, Gui Yang, Xiao-Rui Geng, Jun Liu, Zhi-Gang Liu, Zhanju Liu, Ping-Chang Yang

**Affiliations:** 1 Department of Gastroenterology, the Shanghai Tenth People’s Hospital of Tongji University, Shanghai 200072, China; 2 ENT Institute of Shenzhen University School of Medicine, Shenzhen Key Laboratory of Allergy & Immunology, Shenzhen, 518060, China; 3 Longgang Central Hospital, Shenzhen 518116, China; 4 Shenzhen Women & Children Healthcare Center, Shenzhen, China

## Abstract

The current therapy on allergic inflammation is unsatisfactory. Probiotics improve the immunity in the body. This study aims to test a hypothesis that administration with *Clostridium butyricum (C. butyricum)* enforces the effect of specific immunotherapy (SIT) on intestinal allergic inflammation. In this study, an ovalbumin (OVA) specific allergic inflammation mouse model was created. The mice were treated with SIT or/and *C. butyricum*. The results showed that the intestinal allergic inflammation was only moderately alleviated by SIT, which was significantly enforced by a combination with *C. butyricum*; treating with *C. butyricum* alone did not show much inhibitory efficacy. The increase in the frequency of the interleukin (IL)-10-producing OVA-specific B cell (OVAsBC) was observed in mice in parallel to the inhibitory effect on the intestinal allergic inflammation. The *in vitro* treatment of the OVAsBCs with OVA increased the histone deacetylase-1 (HDAC1) phosphorylation, modulated the transcription of the *Bcl6* gene, and triggered the OVAsBCs to differentiate to the IgE-producing plasma cells. Exposure to both OVA and butyrate sodium in the culture increased the expression of IL-10 in OVAsBCs. In conclusion, administration with *C. butyricum* enforces the inhibitory effect of SIT on allergic inflammation in the mouse intestine.

The aberrant T helper (Th) 2 polarization is one of the major pathological states of chronic intestinal inflammation; such as the food allergen related intestinal inflammation^1^ and a part of the cases of inflammatory bowel disease^2^. Allergic inflammation is featured as an abnormal increase in the allergy-relative cells and cytokines in the local tissue as well as in the peripheral system. The pathogenesis of allergic inflammation has not been fully elucidated yet. Although researches in this area have been advanced rapidly in recent years, the remedies to inhibit allergic inflammation are still limited^3^.

The clinical symptoms of food allergy are initiated by the specific IgE-mediated mast cell activation. The aberrant immune responses result in the production of specific IgE by plasma cells. Mediators from mast cells and eosinophils are the major inflammatory factors to induce allergic inflammation. Thus, to modulate IgE production in plasma cells may suppress the allergic inflammation^4^. After receiving antigen information from Th2 cells, B cells become antigen specific B cells, which may become memory B cells, or further develop into plasma cells with the capacity to produce antigen specific IgE. Yet, the factors leading B cells to become plasma cells are not fully understood.

Specific immunotherapy (SIT) is the only specific remedy to treat allergen related inflammation in the body. SIT has demonstrated the ability to desensitize patients to specific food allergens, which involves administering gradually increasing doses of an allergen over time to induce immunologic changes^5^. The ultimate goal of SIT is to induce immune tolerance to specific allergens by inducing antigen specific immune regulatory cells, such as regulatory T cells (Treg) and regulatory B cells (Breg)^6^. Yet, SIT was shown to desensitize patients; however a long lasting effect of tolerance could not be shown^7^. Upon re-exposure to specific antigens, the Tregs and Bregs are activated to release immune suppressor mediators, such as IL-10 and transforming growth factor-β, to suppress other effector T cell activities, thus to inhibit the allergic inflammation^8^. However, the mechanism of generating antigen specific Tregs and Bregs has not been fully understood yet.

Probiotics are defined as “live microorganisms which, when administered in adequate amounts, confer a health benefit on the host”^9^. Published data suggest that administration of probiotics improves the intestinal immunity^10^. Probiotics may inhibit inflammation and/or activate innate immunity in the intestine, which can be used within therapeutic strategies to restore the host gut microbiota^11^. Probiotics contribute to the maintenance of the intestinal homeostasis via activating Toll like receptor 4^12^. It seems that probiotics benefit the host immunity; yet, the underlying mechanism remains to be further elucidated.

In this study, we treated mice with antigen specific allergic inflammation with SIT and one of the probiotic strains, the *Clostridium butyricum (C. butyricum)*. The results showed that administration of *C. butyricum* markedly enforced the therapy of SIT on the antigen specific allergic inflammation in the intestine. The *C. butyricum*-derived butyrate regulated the signal transduction pathway of IgE production in antigen specific B cells and induced IL-10 expression.

## Results

### *C. butyricum* enforces the effect of SIT on intestinal allergic inflammation

**Figure 1:**
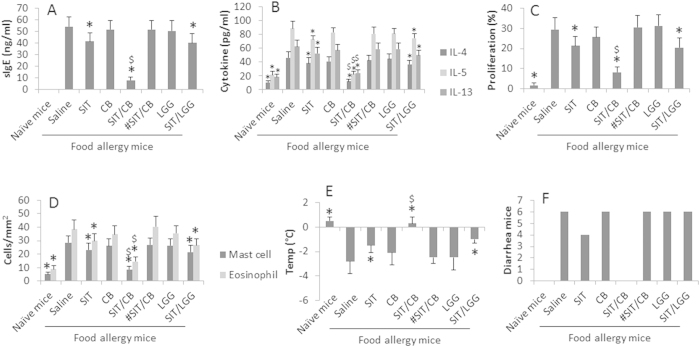
*C. butyricum* promotes therapeutic efficacy on allergic inflammation in the intestine. Food allergy mice were treated with the procedure as denoted on the X axis of the figures and in the text. The data of bars are presented as mean ± SD. *p < 0.01, compared with the “saline” group. $, p < 0.01, compared with SIT group. SIT: Specific immunotherapy. CB: *C. butyricum* (10^9^ organisms/mouse/day by gavage). LGG: *Lactobacillus rhamnosus GG* (10^9^ organisms/mouse/day by gavage). #, IL-10-deficient mice. Each group n = 6. Samples from individual mice were processed separately.

The effect of SIT on food allergen related intestinal inflammation is to be improved^13^. Since probiotics can improve the intestinal immunity^10^, we inferred that probiotics might enforce the effect of SIT on intestinal allergic inflammation. To test the inference, a food allergy mouse model was developed. The allergic mice showed food allergy-like signs in the intestine. The mice were treated with SIT or/and *C. butyricum*. The results showed that SIT moderately improved the allergic inflammation in the intestine, which was markedly enforced by the addition of *C. butyricum*; while treatment with *C. butyricum* alone did not apparently inhibit the intestinal inflammation (Fig. 1). In addition, grouped allergic mice were treated with LGG or SIT/LGG in the same procedures of administration with *C. butyricum* or SIT/*C. butyricum*. The results showed that administration with LGG did not apparently improve the allergic inflammation in the mice (Fig. 1). The data suggest that using *C. butyricum* can enforce the effect of SIT on intestinal allergic inflammation.

### *C. butyricum* plays an important role in the SIT-induced regulatory B cells

**Figure 2:**
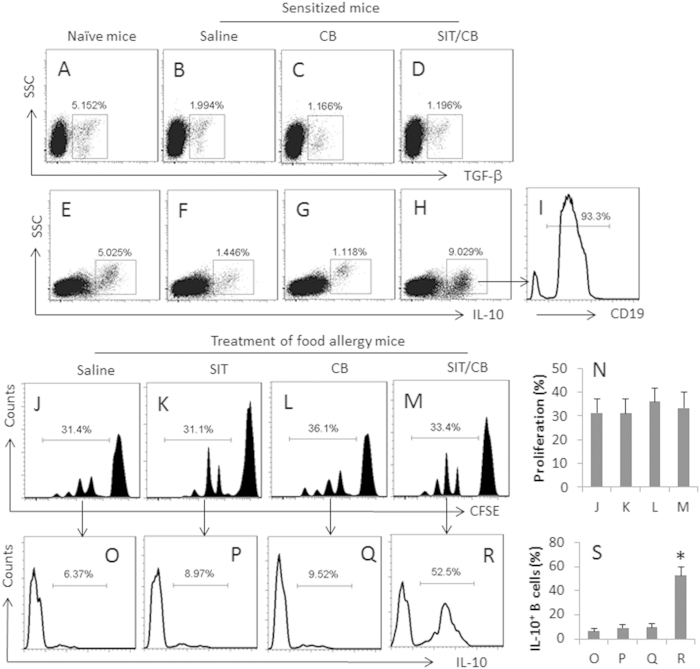
Co-administration with SIT and *C. butyricum* promotes generation of Bregs. Food allergy mice (6 mice per group) were treated with the procedures as denoted above each subpanel of (**A**–**F**). Intestinal lamina propria mononuclear cells (LPMC) were isolated and analyzed by flow cytometry. (**A**–**H**) The dot plots indicate the frequency of TGF-β^+^ cells (**A**–**D**) and IL-10^+^ cells (**E**–**H**). (**I**) The histogram shows the frequency of CD19^+^ cells in the gated cells of panel **H**. (**J**–**M**) CD19^+^ B cells were isolated from LPMCs, labeled with CFSE and cultured in the presence of the specific antigen, OVA (5 μg/ml) and anti-CD40 (20 ng/ml; to prevent B cell apoptosis) for 3 days. The histograms show proliferation of the B cells. (**N**) The bars indicate the summarized data of (**J**–**M**). (**O–R**) The histograms indicate the frequency of IL-10^+^ cells in the proliferating B cells of (**J**–**M**). (**S**) the bars indicate the summarized data of (**O**–**R**). The data of bars in panel S are presented as mean ± SD. *p < 0.01, compared with the group (**O**). The data are representatives of 3 independent experiments.

The data of Fig. 1 imply that the administration of SIT/*C. butyricum* might induce immune tolerant cells, such as Tregs or/and Bregs, in the intestine. We next assessed the immune tolerant mediator TGF-β- and IL-10-positive cells in the isolated LPMCs. The results showed that the frequency of TGF-β positive cells was only slightly increased (Fig. 2A–D) while the treatment with SIT/*C. butyricum* markedly increased the frequency of IL-10 positive cells in LPMCs, which did not occur in mice treated with either SIT alone or *C. butyricum* alone (Fig. 2E–H). We also detected that more than 90% IL-10^+^ cells (Fig. 2H) were CD19 positive (Fig. 2I), The results indicate that administration of SIT/*C. butyricum* induces IL-10-producing B cells in the intestine of allergic mice.

Published data indicate that the IL-10 producing regulatory B cells play a critical role in the inhibition of non-IgE mediated food allergy^14^. We wondered if the IL-10-producing B cells were OVA-specific or *vice versa*. To this end, we isolated CD19^+^ B cells from LPMCs and analyzed by flow cytometry. The results showed that, after exposure to OVA in the culture, the B cells from the allergic mouse intestine proliferated markedly, including 31.4% in allergic mice treated with saline (the control), 31.1% in mice treated SIT and 36.1% in mice treated with *C. butyricum* and 33.4% in mice treated with SIT/*C. butyricum* (Fig. 2J–N). The results indicate the sensitization induces OVAsBCs in the intestine. We next assessed the IL-10^+^ B cells in the proliferated portion of the OVAsBCs. The results showed that about 50% IL-10^+^ B cells were detected in mice treated with SIT/*C. butyricum*, less than 10% of the IL-10^+^ B cells were detected in the rest three groups (Fig. 2O–S). The results indicate that treating allergic mice with SIT/*C. butyricum* induces IL-10^+^ producing B cells (Breg) in the mice. To test if the IL-10 from the Bregs played a critical role in the alleviation of the allergic inflammation in the intestine induced by SIT/*C. butyricum*, in separate experiments, IL-10-deficient mice were sensitized to OVA and treated with SIT/*C. butyricum*; the results showed that using IL-10-deficient mice abolished the therapeutic effect (Fig. 1A–F).

### *C. butyricum*-derived butyrate switches the plasma cell differentiation of OVAsBC to Breg development

**Figure 3:**
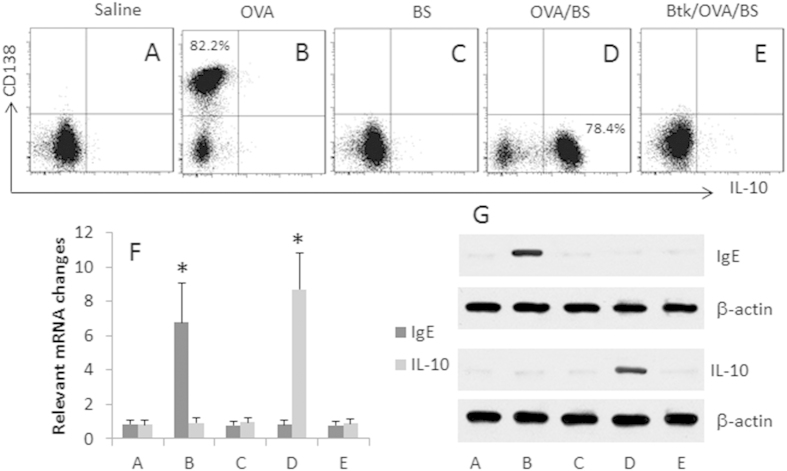
Butyrate modulates OVAsBC differentiation. OVA-specific B cells (OVAsBC) were isolated from the spleen of OVA-sensitized mice with the OVA-tetramer. The OVAsBCs were treated with the procedures as denoted above the dot plots of (**A**–**E**) for 4 days. The cells were analyzed by flow cytometry. (**A**–**E**) The dot plots show the frequency of 138^+^ B cells and IL-10^+^ B cells. (**F**,**G**) The B cells treated with the procedures of (**A**–**E**) were analyzed by RT-qPCR and Western blotting. (F) the bars show the mRNA levels of IgE and IL-10 in the B cells (mean ± SD. *p < 0.01, compared with group **A**). (**G**) The blots show the protein levels of IgE and IL-10 in the B cells. Btk: A BCR signal blocker (PCI-3276533, 1 μM). The data are representative of 3 independent experiments.

To take an insight into the mechanism by which SIT/*C. butyricum* converted OVAsBCs to Bregs in allergic mice, we assessed the effect of butyrate, a product of *C. butyricum*^15^, on the differentiation of OVAsBC. We isolated OVAsBC from the spleen of OVA-sensitized mice. The B cells were cultured with OVA (1 μg/ml; the specific antigen) or/and butyrate sodium for 4 days. As shown by flow cytometry data, in the B cells exposed to OVA alone, a portion of the B cells became CD138 positive (Fig. 3A,B), suggesting the OVAsBCs have differentiated into plasma cells. Exposure to butyrate alone did not induce either CD138^+^ cells or IL-10^+^ B cells (Fig. 3C). In those cells exposed to both OVA and butyrate, about 78.4% of the cells became IL-10 positive (Fig. 3D). The results suggest that, in the presence of specific antigens, butyrate switches antigen specific B cells from the propensity of differentiation to plasma cells to the Breg development. To test the inference, we added Btk, an inhibitor of B cell receptor signal, to the culture in the presence of both OVA and butyrate. Indeed, the generation of IL-10^+^ B cell was abolished (Fig. 3E). The results of Fig. 3A–E were further supported by the data of mRNA and protein analysis with the B cell extracts (Fig. 3F–G).

### Butyrate inhibits HDAC1 activation and promotes Bcl6 expression in OVAsBCs

**Figure 4:**
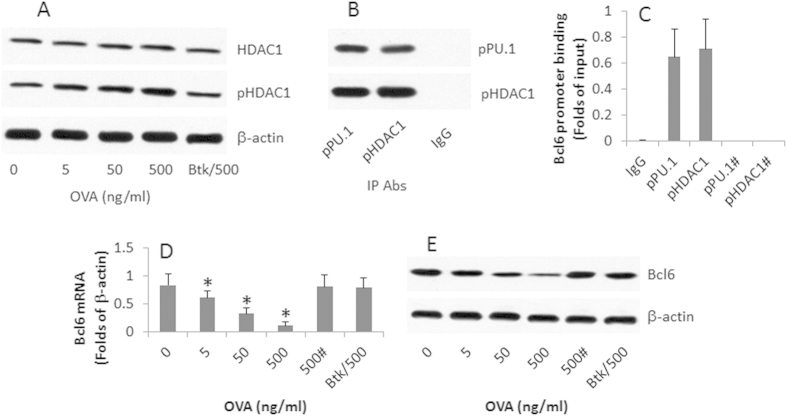
Specific antigens suppress Bcl6 expression in OVAsBCs. OVAsBCs were isolated from the spleen of the OVA-sensitized mice. The cells were stimulated with OVA in the culture for 4 days. The cell extracts were prepared. (**A**) The Western blots indicate the levels of pHDAC1. Btk: A BCR signal blocker (PCI-3276533, 1 μM). (**B**) The Western blots indicates a complex of pHDAC1 and PU.1. (**C**) The bars indicate the Bcl6 promoter binding by pPU.1 or pHDAC1. The ChIP antibodies are denoted on the X axis. #: Butyrate sodium (10 μg/ml) was added to the culture medium. (**D**) The bars indicate the Bcl6 mRNA levels in the OVAsBCs. (**E**) The Western blots indicate the protein levels of Bcl6 in OVAsBCs. The data are representatives of 3 independent experiments.

The data in Fig. 3 suggest that butyrate plays an important role in the conversion of OVAsBC to Bregs during SIT. Since butyrate is an inhibitor of HDAC1^16^, HDAC1 might be involved in Breg generation induced by OVA/butyrate in Fig. 3. To test the inference, we assessed the HDAC1 phosphorylation in the OVAsBCs after exposure to OVA. The results showed that exposure to OVA markedly increased the HDAC1 phosphorylation (Fig. 4A). We also detected a complex of pHDAC1 and PU.1 (the transcription factor of Bcl6) in the OVAsBCs (Fig. 4B). After exposure to OVA, this complex bound the Bcl6 promoter (Fig. 4C) and reduced the Bcl6 gene transcription, which was prevented by the presence of butyrate sodium (Fig. 4D,E). The results suggest that exposure to a specific antigen can induce activation of HDAC1, which is associated with the suppression of Bcl6 expression in OVAsBCs, and did not occur when the OVA was replaced by bovine serum albumin (data not shown). The data imply that the B cell receptor (BCR) activation is involved in the specific antigen-suppressed Bcl6 expression in OVAsBCs. To test the inference, we added a BCR signal blocker, Bruton’s tyrosine kinase (Btk) blocker, to the culture medium. OVA-induced Bcl6 suppression was abolished (Fig. 4A,D,E).

**Figure 5:**
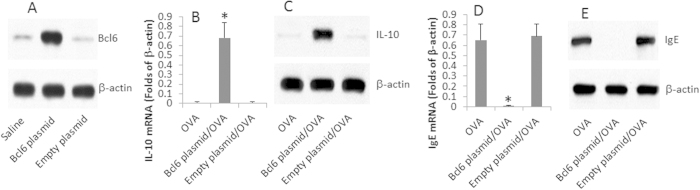
Overexpression of Bcl6 induces IL-10 expression in OVAsBCs. (**A**) A plasmid of Bcl6 was constructed and transfected into OVAsBCs. The Western blots indicate the Bcl6 overexpression in OVAsBCs. (**B**,**C**) The cell extracts were prepared from the OVAsBCs. The bars indicate the levels of IL-10 mRNA expression in OVAsBCs (**B**); the Western blots indicate the protein levels of IL-10 (**C**). (**D**,**E**) The bars indicate the IgE mRNA levels (**D**), the Western blots indicate the IgE protein levels (**E**) in OVAsBCs. The data are representatives of 3 independent experiments.

The above results implicate that Bcl6 may be the molecule related to the expression of IL-10 in OVAsBCs after treating with OVA and butyrate. To test the hypothesis, we overexpressed Bcl6 in OVAsBCs (Fig. 5A). After exposure to OVA in the culture for 4 days, the Bcl6 overexpressing OVAsBCs showed the ability to produce IL-10 (Fig. 5B,C) while the IgE production is below the detectable levels (Fig. 5D,E).

## Discussion

To suppress the allergic inflammation is of significance in the alleviation of the food allergen related intestinal diseases, such as food allergy. The present data suggest that the combination of SIT and *C. butyricum* resulted in a much better inhibitory effect on OVA-specific allergic inflammation in the mouse intestine than using either SIT alone or *C. butyricum* alone. The treatment also induced OVA-specific IL-10-producing B cells (Breg) in the intestine.

SIT is the only specific therapeutic remedy for allergic diseases currently. The efficiency of SIT is supposed to be improved. The present data showed although the SIT alone did suppress the Th2 response in the mice, the clinical symptoms, including the drop in core temperature and diarrhea, were not appreciably improved. The results are consistent with the report from Dr. Berin’s laboratory^13^. Based on published data that using specific probiotic strains can alleviate allergic disorders^17^, we combined SIT and *C. butyricum* in the treatment of food allergy mice. Such a strategy markedly enhanced the therapeutic effect on the allergic inflammation in the intestine. It is to be noted that utilizing *C. butyricum* alone did not apparently alleviate the allergic inflammation in the intestine as observed in the present study. Similar results were reported by Kukkonen *et al.* that probiotic treatment did not reduce the incidence of IgE-associated diseases in children^18^. Not much positive therapeutic outcome induced by oral supplementation with probiotics alone has been reported^19^,^20^.

The present data shed new light on the treatment of food allergy by a combination of SIT with *C. butyricum*, which significantly inhibited food allergen related inflammation in the mouse intestine. A fraction of IL-10-producing antigen specific Breg was detected in the intestine of mice treated with SIT/*C. butyricum*. The results suggest that the therapeutic effect may be generated by IL-10. Data obtained from the experiments using IL-10-deficient mice further support our hypothesis that IL-10 plays an essential role in the generation of this therapeutic effect. Previous reports also showed that IL-10-producing B cells played a critical role in suppressing the non-IgE mediated milk allergy^14^.

Antigen specific B cells can differentiate to either IgE-producing plasma cells, or Bregs. Thus, to elucidate the mediating factors is of significance. The present *in vitro* results showed that exposure to specific antigens in the culture drove OVAsBCs to differentiate into the IgE-producing plasma cells while exposure to OVA/butyrate induced Bregs. The results suggest that butyrate plays a critical role in the induction of Bregs. Since using butyrate alone did not show the ability to convert OVAsBCs to Bregs, the conversion must have been satisfied by additional factors. Indeed, activation of BCR is also needed in the conversion of Bregs. The data are enforced by inhibiting the BCR signal in the presence of OVA/butyrate, in which the signal transduction pathway was disrupted by the presence of Btk, a blocker of BCR signal.

HDAC1 is one of the enzymes in regulating gene transcription^21^. We found that, upon exposure to the specific antigen OVA, HDAC1 was activated and formed a complex with PU.1, a transcription factor of Bcl6; the complex bound to the Bcl6 promoter and inhibited the *Bcl6* gene transcription. Bcl6 has the immune tolerogenic ability^22^. The data suggest that because of the suppression of Bcl6, the OVAsBCs differentiated to IgE-producing plasma cells. The subsequent data supported this inference by showing that overexpression of Bcl6 markedly increased the expression of IL-10 in the OVAsBCs.

In addition to administration with *C. butyricum*, we also observed the effect of LGG on the allergic inflammation in the intestine. The results showed that administration with LGG did not show improvement on the allergic inflammation. Previous reports indicate that probiotics prevent respiratory tract infections and the conventional protection against GI infections^23^; however, the effect of probiotics on food allergy has not been well defined yet. It was reported that probiotic treatment compared with placebo showed no effect on the cumulative incidence of allergic diseases^18^; Rose *et al.* reported that in young children with recurrent wheeze and an atopic family history, oral LGG had no clinical effect on atopic dermatitis or asthma-related events, and only mild effects on allergic sensitization^24^, although probiotics were somewhat effective in preventing dermatitis^25^.

Apart from Bregs, Tregs are also a major type of immune regulatory cells in the body. Type 1 regulatory T (Tr1) cells also produce IL-10. Whether the administration of SIT and *C. butyricum* also up regulates the development of Tr1 cells in the body is an interesting topic and worth being further investigated.

One of the drawbacks of the oral immunotherapy is the therapeutic effect cannot be lasted long^7^. Whether administration of *C. butyricum* can solve this shortcoming is worth being further investigated.

In summary, the present data suggest that a combination of SIT with *C. butyricum* significantly enforces the therapeutic effect on inhibiting the food allergen related inflammation in the intestine, which can be a novel approach for the treatment of food allergy.

## Materials and Methods

### Reagents

The antibodies of HDAC1, pHDAC1, pPU.1, Bcl6 and IgE were purchased from Proteintech (Wuhan, China). ELISA kits of OVA-specific IgE, IL-4 and IL-13, and the fluorochrome-labeled antibodies of IL-10 and IgE were purchased from Biomart (Shanghai, China). Protein G, chromatin immunoprecipitation kit, a biotinylation kit, Butyrate sodium and ovalbumin (OVA) were purchased from Sigma Aldrich (Shanghai, China). PCI-32765 was purchased from Chem Blink (Shanghai, China). The reagents for real time RT-PCR and Western blotting were purchased from Invitrogen (Shanghai, China). Magnetic bead-conjugated streptavidin and immune cell isolation kits were purchased from Miltenyi Biotech (Shanghai, China). The endotoxin levels in all reagents were detected using the Limulus assay (Limulus amebocyte lysate QCL 1000, Bio Whittaker, Walkersville, MD, USA). The reagents used in this study contained <0.2U endotoxin/10 μg reagents.

### Mice

BALB/c mice and IL-10-deficient mice (6–8 weeks old) were purchased from Shanghai Xinmao Experimental Animal Center (Shanghai, China). The mice were maintained in a pathogen-free environment and allowed to access food and water freely. The using mouse in the present study was approved by the Animal Ethic Committee at Shenzhen University. The methods were carried out in “accordance” with the approved guidelines.

### Development of food allergy mouse model

Following our established procedures^26^, a mouse model of intestinal allergic inflammation was developed. Briefly, Mice were gavage-fed with OVA (1 mg/mouse) and cholera toxin (20 μg/mouse) in 0.3 ml saline once a week for 4 weeks. The mice were challenged with OVA (5 mg/mouse) via gavage on week 5 and sacrificed the next day. The assessment of food allergic inflammation in the mice was carried out following our established procedures that were published previously^27^,^28^.

### Specific immunotherapy (SIT)

Following our established procedures^29^, the sensitized mice were treated with oral SIT commencing one week after the last gavage with cholera toxin/OVA for two weeks. Briefly, the OVA was gavage-fed with the doses of 1 mg (days 1 and 2), 5 mg (days 3 and 4), 10 mg (days 5–7), 25 mg (days 8 and 9), and 50 mg (days 10–14). The OVA was administrated with or without mixing with probiotics (see below).

### Treatment with probiotics

*C. butyricum* (the Shenzhen Kexing Biotech; Shenzhen, China) and *Lactobacillus rhamnosus GG* (LGG; ATCC; Beijing, China) were cultured in BHI medium for 16 h at 37 °C on the day before the experiments. The probiotics were centrifuged at 3000 rpm for 5 min, 10^9^ organisms resuspended in 500 μL PBS (pH 7.4). Either of the probiotics were introduced into the stomach of mice daily using a gavage tube attached to a syringe daily commencing one week after the last gavage with cholera toxin/OVA.

### Construction of an Ag-specific Tetramer

To isolate the OVAsBCs, a tetramer was constructed following our established procedures^26^. Briefly, the biotinylated OVA was incubated with magnetic particle-conjugated streptavidin for 30 min at room temperature. Unconjugated reagents less than 10 kDa were filtered through a filter tube by centrifugation. The OVA tetramers were collected for OVAsBC isolation.

### OVAsBC Isolation

Following our established procedures^29^,^30^, lamina propria mononuclear cells (LPMC) were isolated. and OVA tetramer was added to the LPMCs at 2 μg/ml. The cells were incubated for 30 min and then passed through the columns in the magnetic apparatus provided by Miltenyi Biotech. Cells were collected, washed with acidic phosphate-buffered saline (PBS) (pH 3) to remove the bound OVA on the cell surface, and transferred to RPMI 1640 media for further experiments.

### Assessment of OVAsBC proliferation

The isolated OVAsBCs were labeled with carboxyfluorescein diacetate succinimidyl ester (CFSE) and cultured in the presence of OVA (1 μg/ml; the specific Ag) and 2.5 μg/ml anti-CD40 for 3 days. The frequency of CFSE positive cells was assessed by flow cytometry using the CFSE dilution assay.

### Flow cytometry

Single cells were prepared. For surface staining, cells were stained with indicated fluorochrome-labeled antibodies of interest, or isotype IgG (using as negative staining controls). After washing, cells were fixed and permeabilized, and incubated with fluorochrome-labeled antibodies of interest. The cells were analyzed with a flow cytometer (FACSCanto II, BD Biosciences). The data were analyzed with the software Flowjo. The data from the isotype IgG-staining were used as a gating reference.

### Western blotting

Total proteins were extracted, fractioned by SDS-PAGE (sodium dodecyl sulfate-polyacrylamide gel electrophoresis), and transferred onto a PVDF membrane. After blocking with 5% skim milk for 30 min, the membrane was incubated with the primary antibodies overnight at 4 °C, and followed by incubating with the second antibodies (labeled by peroxidase) for 1 h at room temperature. Washing with TBST (Tris-buffered saline Tween 20) was performed after each incubation. The membrane was incubated with ECL (enhanced chemiluminescence). The results were photographed with a chemiluminescence gel imaging system (UVI, Beijing, China).

### Quantitative real-time RT-PCR (RT-qPCR)

The total RNA was isolated with the TRIzol reagent. A cDNA was synthesized using a reverse transcription kit. PCR was performed in a qPCR device (MiniOpticon, Bio Rad) with the SYBR green PCR Master Mix. The cycle time values of the genes of interest were first normalized with β-actin of the same sample, and then the relative difference between control and each treatment group was calculated and expressed as a relative value. Primers using in the present study include: Bcl6 (acaccaccagcctcttatcc and ggcgagtagatgttgctgtg), IL-10 (ggtgagaagctgaagaccct and tgtctaggtcctggagtcca), IgE (atccagacagtgtgaagggg and tgactgaggttccttgaccc) and β-actin (gtgggaatgggtcagaagga and tcatcttttcacggttggcc).

### Immunoprecipitation

The complexes of pHDAC1 and pPU.1 was detected with immunoprecipitation. For the pre-clearing, the cellular extracts were incubated with protein-G beads for 2 h, and centrifuged for 2 min at 2000 rpm. The supernatant was incubated overnight with anti-pPU.1 or anti-pHDAC1 antibody and protein-G beads. The immunoprecipitates were collected and washed with RIPA buffer (50 mM Tris, pH 7.4, 150 mM NaCl, 1% NP-40, 0.25% sodium deoxycholate, 1 mM Na3VO4, 1 mM NaF and protease inhibitors) and then subjected to Western blotting.

### Chromatin immunoprecipitation (ChIP)

ChIP was performed according to the protocol described previously^31^. OVAsBC extracts were cross-linked with formaldehyde and sonicated. Resulting cell lysates (input) were immunoprecipitated with the respective antibodies (2.5 μg each) of interest. The precipitated protein-DNA complexes were subjected to proteinase treatment. The primers used to confirm the binding of factors to the promoter region of Bcl6 were: Forward, gggttcttagaagtggtgatgc; reverse, cagcaacagcaataatcacctg.

### BcL6 overexpression

The Bcl6 DNA vector was constructed by GeneScript (Nanjing, China). 2 × 10^5^ OVAsBCs were isolated from the spleen of OVA-sensitized mice and seeded in each well of a 24-well tissue culture plate 1 day before transfection. The equimolar amount of the Bcl6 DNA vector was transfected into each well, according to the Lipofectamine 2000 Transfection Reagent manual from Invitrogen. The Bcl6 expression in the OVAsBC was assessed by Western blotting.

### Statistics

The data are presented as mean ± SD. The difference between two groups was determined by Student t test or ANOVA if more than three groups. A p < 0.05 was set as a significant criterion.

## Additional Information

**How to cite this article**: Shi, Y. *et al.* Specific immunotherapy in combination with *Clostridium butyricum* inhibits allergic inflammation in the mouse intestine. *Sci. Rep.* **5**, 17651; doi: 10.1038/srep17651 (2015).

## References

[b1] Chu D. K. *et al.* T helper cell IL-4 drives intestinal Th2 priming to oral peanut antigen, under the control of OX40L and independent of innate-like lymphocytes. Mucosal Immunol 7, 1395–1404 (2014). 24781052 10.1038/mi.2014.29

[b2] McNamee E. N. *et al.* Novel model of TH2-polarized chronic ileitis: the SAMP1 mouse. Inflamm Bowel Dis 16, 743–752 (2010). 19856411 10.1002/ibd.21148 PMC3786705

[b3] de Silva D. *et al.* Acute and long-term management of food allergy: systematic review. Allergy 69, 159–167 (2014). 24215577 10.1111/all.12314

[b4] Gauvreau G. M. *et al.* Targeting membrane-expressed IgE B cell receptor with an antibody to the M1 prime epitope reduces IgE production. Sci Transl Med 6, 243ra285 (2014). 10.1126/scitranslmed.3008961 24990880

[b5] Le U. H. & Burks A. W. Oral and sublingual immunotherapy for food allergy. World Allergy Organ J 7, 35 (2014). 25709745 10.1186/1939-4551-7-35 PMC4325942

[b6] Jutel M. & Akdis C. A. T-cell regulatory mechanisms in specific immunotherapy. Chem Immunol Allergy 94, 158–177 (2008). 18802346 10.1159/000155000

[b7] Frati F. *et al.* Mucosal immunization application to allergic disease: sublingual immunotherapy. Allergy Asthma Proc 28, 35–39 (2007). 17390755 10.2500/aap.2007.28.2919

[b8] Palomares O., Martin-Fontecha M. & Lauener R. Regulatory T cells and immune regulation of allergic diseases: roles of IL-10 and TGF-beta. Genes Immun 15, 511–520 (2014). 25056447 10.1038/gene.2014.45

[b9] Saez-Lara M. J., Gomez-Llorente C., Plaza-Diaz J. & Gil A. The Role of Probiotic Lactic Acid Bacteria and Bifidobacteria in the Prevention and Treatment of Inflammatory Bowel Disease and Other Related Diseases: A Systematic Review of Randomized Human Clinical Trials. Biomed Res Int 2015, 505878 (2015). 25793197 10.1155/2015/505878 PMC4352483

[b10] Wang H. *et al.* Are there any different effects of Bifidobacterium, Lactobacillus and Streptococcus on intestinal sensation, barrier function and intestinal immunity in PI-IBS mouse model? PLoS One 9, e90153 (2014). 24595218 10.1371/journal.pone.0090153 PMC3940848

[b11] Benjamin J. L. *et al.* Randomised, double-blind, placebo-controlled trial of fructo-oligosaccharides in active Crohn’s disease. Gut 60, 923–929 (2011). 21262918 10.1136/gut.2010.232025

[b12] Zeuthen L. H., Fink L. N. & Frokiaer H. Toll-like receptor 2 and nucleotide-binding oligomerization domain-2 play divergent roles in the recognition of gut-derived lactobacilli and bifidobacteria in dendritic cells. Immunology 124, 489–502 (2008). 18217947 10.1111/j.1365-2567.2007.02800.x PMC2492941

[b13] Leonard S. A., Martos G., Wang W., Nowak-Wegrzyn A. & Berin M. C. Oral immunotherapy induces local protective mechanisms in the gastrointestinal mucosa. J Allergy Clin Immunol 129, 1579-1587.e1571 (2012). 10.1016/j.jaci.2012.04.009 PMC3367084 22554705

[b14] Lee S. J., Noh G. & Lee J. H. *In Vitro* Induction of Allergen-Specific Interleukin-10-Producing Regulatory B Cell Responses by Interferon-gamma in Non-Immunoglobulin E-Mediated Milk Allergy. Allergy Asthma Immunol Res 5, 48–54 (2013). 23277878 10.4168/aair.2013.5.1.48 PMC3529229

[b15] Zhao X., Guo Y., Liu H., Gao J. & Nie W. Clostridium butyricum reduce lipogenesis through bacterial wall components and butyrate. Appl Microbiol Biotechnol 98, 7549–7557 (2014). 24878750 10.1007/s00253-014-5829-x

[b16] Jiang J. *et al.* Trek1 contributes to maintaining nasal epithelial barrier integrity. Sci Rep 5, 9191 (2015). 25778785 10.1038/srep09191 PMC7365316

[b17] Nermes M., Salminen S. & Isolauri E. Is there a role for probiotics in the prevention or treatment of food allergy? Curr Allergy Asthma Rep 13, 622–630 (2013). 23934549 10.1007/s11882-013-0381-9

[b18] Kukkonen K. *et al.* Probiotics and prebiotic galacto-oligosaccharides in the prevention of allergic diseases: a randomized, double-blind, placebo-controlled trial. J Allergy Clin Immunol 119, 192–198 (2007). 17208601 10.1016/j.jaci.2006.09.009

[b19] Hol J. *et al.* The acquisition of tolerance toward cow’s milk through probiotic supplementation: a randomized, controlled trial. J Allergy Clin Immunol 121, 1448–1454 (2008). 18436293 10.1016/j.jaci.2008.03.018

[b20] Brouwer M. L. *et al.* No effects of probiotics on atopic dermatitis in infancy: a randomized placebo-controlled trial. Clin Exp Allergy 36, 899–906 (2006). 16839405 10.1111/j.1365-2222.2006.02513.x

[b21] Moser M. A., Hagelkruys A. & Seiser C. Transcription and beyond: the role of mammalian class I lysine deacetylases. Chromosoma 123, 67–78 (2014). 24170248 10.1007/s00412-013-0441-x PMC3967066

[b22] Linterman M. A. *et al.* Foxp3+ follicular regulatory T cells control the germinal center response. Nat Med 17, 975–982 (2011). 21785433 10.1038/nm.2425 PMC3182542

[b23] Hojsak I. *et al.* Lactobacillus GG in the prevention of nosocomial gastrointestinal and respiratory tract infections. Pediatrics 125, e1171–1177 (2010). 20403940 10.1542/peds.2009-2568

[b24] Rose M. A. *et al.* Efficacy of probiotic Lactobacillus GG on allergic sensitization and asthma in infants at risk. Clin Exp Allergy 40, 1398–1405 (2010). 20604800 10.1111/j.1365-2222.2010.03560.x

[b25] Doege K. *et al.* Impact of maternal supplementation with probiotics during pregnancy on atopic eczema in childhood–a meta-analysis. Br J Nutr 107, 1–6 (2012). 21787448 10.1017/S0007114511003400

[b26] Geng X. R. *et al.* Insulin-like growth factor-2 enhances functions of antigen (Ag)-specific regulatory B cells. J Biol Chem 289, 17941–17950 (2014). 24811165 10.1074/jbc.M113.515262 PMC4067224

[b27] He S. H. *et al.* Interferon-lambda mediates oral tolerance and inhibits antigen-specific, T-helper 2 cell-mediated inflammation in mouse intestine. Gastroenterology 141, 249-258, 258.e241-242 (2011). 10.1053/j.gastro.2011.04.006 21569774

[b28] Feng B. S. *et al.* Disruption of T-cell immunoglobulin and mucin domain molecule (TIM)-1/TIM4 interaction as a therapeutic strategy in a dendritic cell-induced peanut allergy model. J Allergy Clin Immunol 122, 55-61, 61.e51-57 (2008). 10.1016/j.jaci.2008.04.036 18547633

[b29] Zhang H. P. *et al.* TSP1-producing B cells show immune regulatory property and suppress allergy-related mucosal inflammation. Sci Rep 3, 3345 (2013). 24736213 10.1038/srep03345 PMC4002291

[b30] Yang G. *et al.* Insulin-like growth factor 2 enhances regulatory T-cell functions and suppresses food allergy in an experimental model. J Allergy Clin Immunol 133, 1702-1708.e1705 (2014). 10.1016/j.jaci.2014.02.019 24698315

[b31] Lin J., Wang X. & Dorsky R. I. Progenitor expansion in apc mutants is mediated by Jak/Stat signaling. BMC Dev Biol 11, 73 (2011). 22136118 10.1186/1471-213X-11-73 PMC3247185

